# Negative Immunomodulatory Effects of Type 2 Porcine Reproductive and Respiratory Syndrome Virus-Induced Interleukin-1 Receptor Antagonist on Porcine Innate and Adaptive Immune Functions

**DOI:** 10.3389/fimmu.2019.00579

**Published:** 2019-03-26

**Authors:** Teerawut Nedumpun, Navapon Techakriengkrai, Roongroje Thanawongnuwech, Sanipa Suradhat

**Affiliations:** ^1^Interdisciplinary Program in Medical Microbiology, Graduate School, Chulalongkorn University, Bangkok, Thailand; ^2^Department of Veterinary Microbiology, Faculty of Veterinary Science, Chulalongkorn University, Bangkok, Thailand; ^3^Center of Excellence in Emerging Infectious Diseases in Animals, Chulalongkorn University (CU-EIDAs), Bangkok, Thailand; ^4^Department of Veterinary Pathology, Faculty of Veterinary Science, Chulalongkorn University, Bangkok, Thailand

**Keywords:** type 2 PRRSV, pig, immunomodulatory effect, IL-1Ra, interleukin-1 receptor antagonist, innate and adaptive immune response

## Abstract

Impaired innate and adaptive immune responses are evidenced throughout the course of PRRSV infection. We previously reported that interleukin-1 receptor antagonist (IL-1Ra) was involved in PRRSV-induced immunosuppression during an early phase of infection. However, the exact mechanism associated with PRRSV-induced IL-1Ra immunomodulation remains unknown. To explore the immunomodulatory properties of PRRSV-induced IL-1Ra on porcine immune functions, monocyte-derived dendritic cells (MoDC) and leukocytes were cultured with type 2 PRRSV, and the immunological role of IL-1Ra was assessed by addition of anti-porcine IL-1Ra Ab. The results demonstrated that PRRSV*-*induced IL-1Ra reduced phagocytosis, surface expression of MHC II (SLA-DR) and CD86, as well as downregulation of *IFNA* and *IL1* gene expression in the MoDC culture system. Interestingly, IL-1Ra secreted by the PRRSV-infected MoDC also inhibited T lymphocyte differentiation and proliferation, but not IFN-γ production. Although PRRSV-induced IL-1Ra was not directly linked to IL-10 production, it contributed to the differentiation of regulatory T lymphocytes (Treg) within the culture system. Taken together, our results demonstrated that PRRSV-induced IL-1Ra downregulates innate immune functions, T lymphocyte differentiation and proliferation, and influences collectively with IL-10 in the Treg induction. The immunomodulatory roles of IL-1Ra elucidated in this study increase our understanding of the immunobiology of PRRSV.

## Introduction

Porcine Reproductive and Respiratory Syndrome Virus (PRRSV) is one of the major pathogens affecting the pig production industry worldwide. PRRSV is an enveloped, positive-stranded RNA virus, which belongs to the genus *Porartevirus*, family *Arteriviridae* and order *Nidovirales* (1). PRRSV can be classified into two genotypes; type 1 (EU) and type 2 (US) (2), but rapid evolutionary rate often leads to the emergence of PRRSV strain variants (3, 4). Most PRRSV strains have the capacity to impair host immunity, leading to generalized immunosuppression in infected pigs (5). Consequently, PRRSV infection usually increases the severity of other concomitantly infected pathogens (6).

Innate immunity is recognized as a key modulator for induction of efficient anti-viral immune responses (7, 8). However, previous studies strongly evidenced that PRRSV primarily infects innate immune cells, such as macrophage and non-conventional dendritic cells (DC), and suppresses their functions (9–11). PRRSV inhibits maturation of antigen presenting cells (APC) and decreases levels of both MHC II (SLA-DR) and co-stimulatory molecule (CD80 and CD86) expression (12, 13). Moreover, enhanced expression of programed cell death-ligand 1 (PD-L1) on PRRSV-infected APC supported induction of cell apoptosis and regulatory T lymphocyte (Treg) differentiation (14, 15). During an early phase of PRRSV infection, production of type I IFN and pro-inflammatory cytokines (IL-1, IL-6, and TNF-α) was drastically suppressed, leading to uncontrolled viral replication (16, 17). It has been suggested that PRRSV-induced suppression of innate immunity potentially causes poor adaptive immune responses (18), characterized by attenuated T lymphocyte proliferation, and poor induction of PRRSV-specific IFN-γ-producing cells (19, 20) together with delayed neutralizing antibody responses (21).

The negative immunomodulatory mechanism induced by type 2 PRRSV has been linked to the induction of interleukin-10 (IL-10), which in turn provides an immunological niche for Treg expansion (18, 22). However, the immunosuppressive effect of PRSSV might not be solely associated with the induction of IL-10 and Treg as these factors develop slowly, ~1 week after the suppression of innate immunity (23, 24). Moreover, levels of IL-10 and Treg induction were different among the PRRSV strains (25).

Recently, interleukin-1 receptor antagonist (IL-1Ra) induced by PRRSV was suggested to play an important role during the early phase of PRRSV infection as increased levels of IL-1Ra were observed both *in vitro* and in PRRSV-infected pigs (26). Although effect of IL-1Ra on porcine immune responses remains elusive, several evidences have been shown in both human and mouse models. IL-1Ra competitively binds to IL-1 receptor (IL-1R), and subsequently inhibits IL-1-induced signaling cascades (27). Interestingly, APC maturation and induction of type I IFN, IL-1, and TNF-α were shown to be modulated by IL-1Ra production (27–29). Blocking the IL-1R signaling pathway could inhibit antigen-specific T lymphocyte activation and proliferation (29, 30). In addition, immunomodulatory effects of IL-1Ra were also reported in the progression of some infectious diseases. Increased level of IL-1Ra production induced by *Yesinia pestis* could suppress pro-inflammatory cytokine production, resulting in prolonged bacterial survival during the early stage of infection (31). Likewise, human immunodeficiency virus (HIV)-induced IL-1Ra production weakens inflammatory processes through inhibition of IL-1 synthesis in human monocytes (32). Altogether, these evidences strongly indicate that IL-1Ra represents a key immunomodulator during an early phase of immune responses. In this study, the impact of PRRSV-induced IL-1Ra on porcine innate and adaptive immune functions were investigated.

## Materials and Methods

### Viruses and Cells

Type 2 PRRSV strain 01NP1 (33) and classical swine fever virus (CSFV) were kindly provided by Chulalongkorn University Veterinary Diagnostic Laboratory (CU-VDL; Bangkok, Thailand). PRRSV and CSFV were cultured and titrated in MARC-145 (CU-VDL) and SK6 (CU-VDL) cell lines, respectively. Mock-infected cell lysates were prepared from MARC-145 (for PRRSV) and SK6 (for CSFV) cells, respectively. All viruses and mock-infected cell lysates were stored at −80°C until needed.

### Antibodies

Anti-PRRSV N mAb (SDOW-17, IgG) was purchased from RTI (SD, USA). Anti-porcine SLA-DR mAb (1053H2-18, IgG2a), anti-porcine CD3-FITC mAb (BB23-8E6, IgG2b), biotinylated anti-porcine CD4 mAb (74-12-4, IgG2b), and anti-porcine CD8-PE mAb (76-2-11, IgG2a) were purchased from Southern Biotech (Birmingham, AL, USA). Biotinylated anti-porcine IFN-γ mAb (P2C11) was purchased from BD Biosciences (San Jose, CA, USA). Anti-porcine IL-10 mAb (945A4C437B1, IgG1) was purchased from Biosource (Camarillo, CA, USA). Anti-human FOXP3 mAb-APC (236A/E7, IgG1) was purchased from eBioscience (SanDiego, CA, USA). Anti-porcine CD25 mAb (K231.3B2, IgG1), goat anti-mouse IgG1-FITC and goat anti-mouse IgG2a-FITC were purchased from AbD Serotec (Kidlington, UK). Anti-human CD86-PEcy7 mAb (IT2.2, IgG1), anti-BrdU-FITC (3D4, IgG1), streptavidin-APC and streptavidin-PEcy7 were purchased from BioLegend® (San Diego, CA, USA). Goat anti-mouse IgG1-Alexaflur 647 and streptavidin-PE were purchased from ThermoFisher Scientific (Invitrogen, Carlsbad, CA, USA).

### Isolation of Porcine Leukocytes and Generation of Monocyte-Derived Dendritic Cells (MoDC)

Crossbred, PRRSV-seronegative pigs were previously immunized with CSFV-modified live vaccine (MLV) (COGLAPEST®, Ceva Santé Animale, Libourne, France) at 4 and 7 weeks of age. At 16 weeks of age, porcine peripheral blood mononuclear cells (PBMC) were isolated from heparinized whole blood by density gradient centrifugation, using LymphoSep™ (MP Biomedicals, California, USA) according to the manufacturer's procedure. MoDC were generated as previously described (34). Briefly, the PBMC were resuspended at 5 x 10^6^ cells/mL in Iscove's Modified Dulbecco's Media (IMDM) (GIBCO, Carlsbad, CA, USA), and incubated at 37°C and 5% CO_2_ for 2 h. Non-adherent cells, referred as peripheral blood lymphocytes (PBL), were collected and stored at 5 × 10^7^ cells/mL in liquid nitrogen until needed. The remaining adherent cells were cultured with 10 ng/mL porcine recombinant IL-4 (R&D system, Minneapolis, MN, USA) and 25 ng/mL porcine recombinant GM-CSF (R&D system) for 7 days. For downstream experiments, PBMC, PBL, and MoDC were plated in complete RPMI, containing advanced RPMI (GIBCO), 10% FBS (GIBCO), 2 mM L-glutamine (GIBCO), antibiotic/antimycotic solution (GIBCO), 25 mM HEPES (GIBCO), and 50 μM β-mercaptoethanol (Sigma Chemical Co., St. Loius, USA).

### *In vitro* IL-1Ra Neutralization Assay

#### On MoDC

MoDC (1 × 10^6^ cells/200 μl/well) were incubated with 0.1 m.o.i. of type 2 PRRSV or mock (MARC-145 cell lysate) in 24-well plates at 37°C and 5% CO_2_. In some experimental conditions, cells were pre-treated with final concentration of 10 ng/mL polyclonal goat anti-porcine IL-1Ra antibody (R&D system, clone AF780) or polyclonal goat IgG isotype control antibody (R&D system) at 2 h post-inoculation to neutralize PRRSV-induced IL-1Ra which was then cultured for another 22 h. To determine the effect of PRRSV-induced IL-1Ra on MoDC phagocytic activity, the antibody pre-treated MoDC (2 × 10^6^ cell) were further incubated with inactivated *E. coli*-FITC (ThermoFisher Scientific) in complete RPMI at a MoDC:*E. coli* ratio of 1:50 for 10 min at 37°C. Immediately after, cold PBSA was added to stop the phagocytic activity. The cell pellet was washed twice with PBSA and subjected to flow cytometric analyses. To measure the effect of PRRSV-induced IL-1Ra on MoDC maturation and cytokine gene expression, 1 μg/mL of LPS (Sigma Chemical Co.) was added into each respective well at 24 h post-inoculation, and cultured for another 24 h. Afterward, the cells were harvested and subjected to immunofluorescent staining and mRNA extraction.

#### On T Lymphocyte Responses

The supernatants from mock or PRRSV-infected MoDC at 24 h post-inoculation were incubated with 10 ng/mL of either anti-porcine IL-1Ra or isotype control antibodies for 2 h for IL-1Ra neutralization. Then, PBMC or PBL (2 × 10^6^ cells/well) were incubated with 200 μL of these antibody pre-treated supernatants prior to performing downstream functional assays. To determine the effects of PRRSV-induced IL-1Ra on T lymphocyte differentiation, the supernatant-treated PBMC were inoculated with 0.1 m.o.i. of CSFV or mock (SK6 cell lysate) at 37°C and 5% CO_2_. The cell pellets were collected at 0, 12, 24, 48, and 72 h post-inoculation for mRNA extraction.

To determine effects of PRRSV-induced IL-1Ra on IFN-γ production and proliferation, the pre-treated PBL or PBMC were incubated with 1 μg/mL PHA (Sigma Chemical Co.), DMSO (Sigma Chemical Co.), 0.1 m.o.i. of CSFV or mock (SK6 cell lysate) for 48 (IFN-γ production) or 96 h (proliferation). For the proliferation assay, the cells were cultured with 10 μM Bromodeoxyuridine (BrdU, BioLegend®) prior to incubation with indicated treatments. The cells were harvested and subjected to immunofluorescent staining and flow cytometric analyses.

#### On IL-10-Producing Cells and Treg

PBMC (2 × 10^6^ cells/well) were inoculated with 0.1 m.o.i. of type 2 PRRSV or mock (MARC-145 cell lysate) in 24-well plates at 37°C and 5% CO_2_ for 2 h. The cultures were subsequently treated with anti-porcine IL-1Ra or isotype control antibodies (10 ng/mL) and incubated for another 46 h. The cells were harvested and subjected to immunofluorescent staining for IL-10-producing T lymphocytes and Treg.

### IL-1Ra ELISA

PBMC (2 × 10^6^ cells/well) or MoDC (1 × 10^6^ cells/well) were cultured with 0.1 m.o.i. of type 2 PRRSV or mock (MARC-145 cell lysate) in 24-well plates at 37°C and 5% CO_2_ for 48 h. The culture supernatants were collected and measured for the level of PRRSV-induced IL-1Ra by porcine IL-1Ra ELISA (CUSABIO, Wuhan, China).

### Immunofluorescent Staining and Flow Cytometric Analyses

To confirm PRRSV infection, PBMC and MoDC were stained and permeabilized with 1:100 anti-PRRSV N mAb (SDOW-17) diluted in Reagent B (Leucoperm, AbD serotec) in the dark at the 4°C for 30 min followed by PBSA washing. Subsequently, 1:100 of goat anti-mouse IgG1-FITC mAb diluted in PBSA supplemented with 0.5% BSA and 0.1% sodium azide, referred as the FACS buffer, were added and incubated in the dark at 4°C for 30 min. The stained cells were subjected to flow cytometric analyses.

After performing the neutralization assays, the cells (1 × 10^6^ cells/well) were harvested and transferred into 96-well round-bottom plates and then washed twice with FACs buffer. For immunofluorescent staining of surface molecules including SLA-DR, CD86, CD3, CD4, CD8, and CD25, primary mAbs at indicated concentration; 1:100 of anti-SLA-DR, 1:50 of anti-CD86-PEcy7, 1:50 of anti-CD3-FITC, 1:50 of biotinylated anti-CD4, 1:50 of anti-CD8-PE, or 1:100 of anti-CD25 mAbs, diluted in FACS buffer at final volume 50 μL/reaction, were added and further incubated in the dark at 4°C for 30 min. For secondary staining, 1:500 of streptavidin-PE, 1:500 of streptavidin-PEcy7, 1:100 of goat anti-mouse IgG1-FITC or 1:100 of goat anti-mouse IgG2a-FITC, diluted in FACS buffer was added to the cells and incubated in the dark at 4°C for 30 min.

For intracellular staining, the cells were then fixed and permeabilized with 50% reagent A (Leucoperm, Serotec), diluted in FACS buffer, for 30 min. For primary staining, 1:100 of anti-BrdU-FITC, 1:100 of biotinylated anti-IFN-γ, 1:100 of IL-10 (IgG1) or 1:20 of anti-FOXP3-APC mAbs, diluted in Reagent B (Leucoperm, Serotec) was added to the cells and further incubated in the dark at 4°C for 45 min. For secondary staining, 1:500 of streptavidin-APC or 1:100 of goat anti-mouse IgG1-Alexaflur 647, diluted in FACS buffer was added to the cells and incubated in the dark at 4°C for 30 min.

Cells, stained with the different isotype controls, were used to set the background cut-off of the study. The fluorescent minus one (FMO) staining samples were also performed during the establishment and validation of the assay. The cells were gated at least 1 × 10^5^ cell/events for each analysis. Flow cytometric analyses were performed using FC 500 MPL (Beckman Coulter, CA, USA).

### Quantitative Polymerase Chain Reaction (qPCR)

Total mRNAs were isolated from the cells (2 × 10^6^ cells/reaction) by using the total mRNA extraction kit (Biotechrabbit, Germany) according to the manufacturer's instruction. The extracted mRNAs were assayed by NanoDrop (Thermo scientific, USA) and converted to cDNA using a cDNA synthesis kit (Invitrogen, USA). Levels of porcine *IFNA, IL1, IL6, TBET, GATA3, RORGT, FOXP3*, and *GAPDH* expression were quantified by SYBR green-based qPCR using the specific primer sets shown in Table 1. qPCR reaction was carried out as previously described (26, 35–37). Ct values of each gene were normalized against the housekeeping gene; *GAPDH*. Differences in Ct values between the treatment groups were analyzed by the formula 2^−ΔΔCt^.

**Table 1:** Sequences of the qPCR primers.

**Gene**	**NCBI Accession no**.	**Primer sequence (5^′^> 3^′^)**	**Product size (bp)**	**References**
*IFNA*	XM_003480507.3	F: CTG-GAG-GAG-GAC-TCC-AT	268	(26)
		R: GAG-TCT-GTC-TTG-CAG-GTT		
*IL1B*	NM_214055.1	F: AAC-GTG-CAA-TGA-TGA-CTT-TG	292	(26)
		R: CAC-TTC-TCT-CTT-CAA-GTC-CC		
*IL6*	JQ839263.1	F: AGA-ACT-CAT-TAA-GTA-CAT-CCT-CG	180	(34)
		R: AGA-TTG-GAA-GCA-TCC-GTC		
*TBET*	XM_003132081.4	F: TCA-ATC-CTA-CTG-CCC-ACT-AC	151	(35)
		R: TTA-GGA-GAC-TCT-GGG-TGA-AC		
*GATA3*	XM_022745494.1	F: ACA-GAC-CCC-TGA-CCA-TGA-AG	193	(35)
		R: GGA-GAT-GTG-GCT-GAG-AGA-GG		
*FOXP3*	XM_021079539.1	F: CTC-CTA-CTC-CCT-GCT-GGC-AAA-T	283	(24)
		R: TAC-AAT-ACA-GCA-GGA-ACC-CTT-GTC-A		
*GAPDH*	XM_005658673.2	F: AAG-TGG-ACA-TTG-TCG-CCA-TC	318	(24)
		R: TCA-CAA-ACA-TGG-GGG-CAT-C		

### Statistical Analyses

Data were analyzed using student *t*-test or analysis of variance (ANOVA) followed by Tukey's multiple comparison tests. All statistical analyses were performed using GraphPad Prism for Windows (GraphPad Software Incorporated, San Diego, CA, USA).

## Results

### PRRSV-Induced IL-1Ra Impaired Phagocytic Activity, Maturation and Innate Cytokine Productions

**Figure 1:**
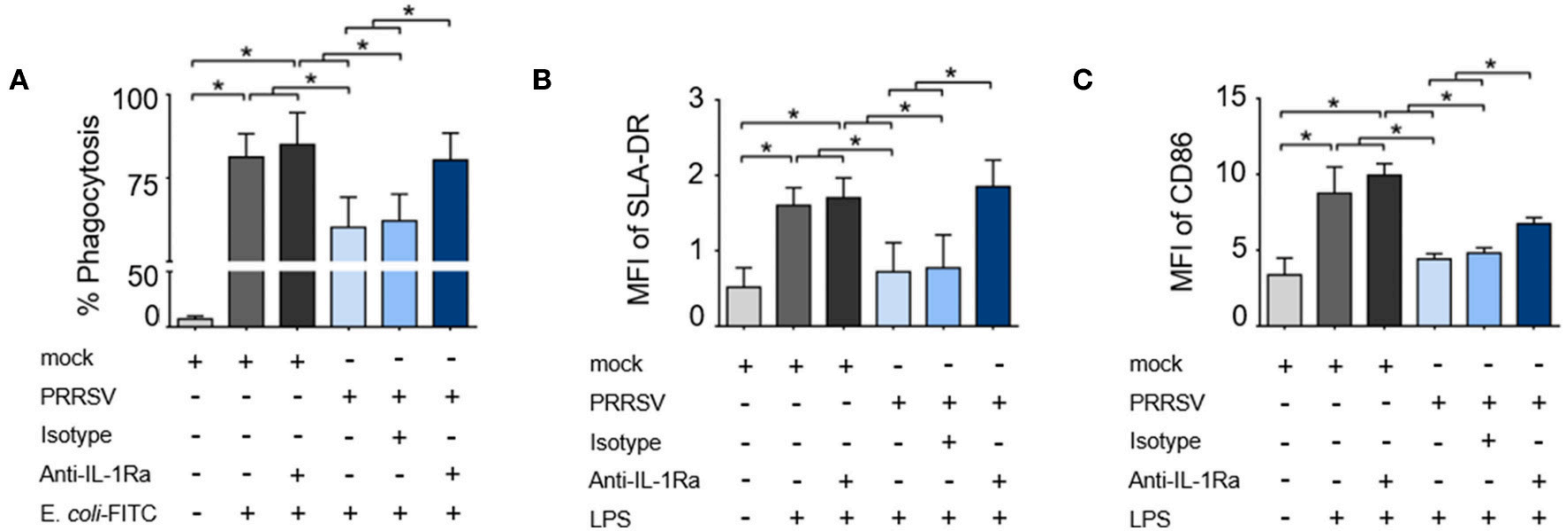
PRRSV-induced IL-1Ra inhibited porcine innate immune functions. PRRSV-induced IL-1Ra inhibited **(A)** phagocytic activity, **(B)** SLA-DR and **(C)** CD86 expression. MoDC were cultured with type 2 PRRSV or mock, in the presence of anti-IL-1Ra Ab. LPS was added into the culture and further incubated for 24 h. ± indicates presence/absence of indicated treatment within the culture. Data represents mean ± SD from 5 pigs. Statistical significance was analyzed using ANOVA followed by Tukey's test. * indicates significant difference at *p* < 0.05.

To investigate the role of PRRSV-induced IL-1Ra on porcine innate immune functions, MoDC were infected with type 2 PRRSV or mock (MARC-145 cell lysate) and subsequently cultured with anti-porcine IL-1Ra Ab. Antibody preincubation had no effect on the numbers of PRRSV-infected cells (Supplementary Figure 1A). Significant IL-1Ra production was observed in the supernatants of PRRSV-infected MoDC as confirmed by ELISA (Supplementary Figure 1B). PRRSV infection significantly decreased phagocytic activity of MoDC, which could be restored by the neutralization of IL-1Ra (Figure 1A and Supplementary Figure 2A). Addition of anti-IL-1Ra Ab alone did not affect the phagocytic activity of MoDC. Next, we investigated the effect of PRRSV-induced IL-1Ra on MoDC maturation. Consistent with previous findings (38–40), SLA-DR and CD86 expressions on LPS-induced MoDC were significantly decreased by PRRSV. The blockade of IL-1Ra in the culture increased SLA-DR (MHC II) and restored CD86 expression (Figures 1B,C and Supplementary Figures 2B,C).

**Figure 2:**
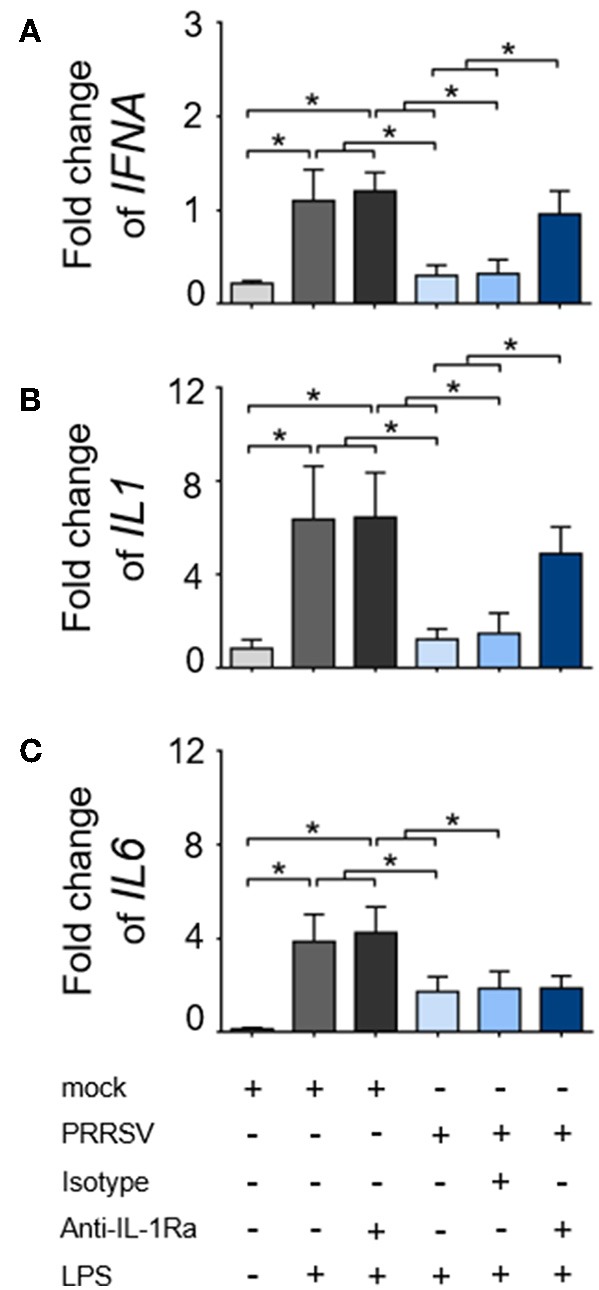
PRRSV-induced IL-1Ra inhibited **(A)** *IFNA* and **(B)** *IL1*, **(C)** but not *IL6* expression. MoDC were cultured with type 2 PRRSV or mock, in the presence of anti-IL-1Ra Ab. LPS was added into the culture and further incubated for 24 h. ± indicates presence/absence of indicated treatment within the culture. Data represents mean ± SD from 5 pigs. Statistical significance was analyzed using ANOVA followed by Tukey's test. * indicates significant difference at *p* < 0.05.

As downregulation of *IFNA, IL1*, and *IL6* expression was always reported to precede the IL-10 induction during the course of PRRSV infection (17, 41–43), we hypothesized that these effects were mediated by PRRSV-induced IL-1Ra. In response to LPS stimulation, the expressions of *IFNA, IL1*, and *IL6* genes in the cultured MoDC were significantly increased and PRRSV abolished these innate cytokine gene expressions (Figures 2A–C). Neutralization of IL-1Ra restored *IFNA* and *IL1* gene expressions up to the levels observed in the control treatments (mock). However, neutralization of IL-1Ra appeared to have little effect on the level of *IL6* gene expression (Figures 2A–C) suggesting that PRRSV downregulated *IL6* by a different mechanism. Altogether, these findings indicated that PRRSV inhibited several key functions of APC, including phagocytic activity, antigen processing and presentation and certain pro-inflammatory cytokine production through IL-1Ra production.

### PRRSV-Induced IL-1Ra Altered Expressions of Helper T Lymphocyte Transcriptional Factors During CSFV Reactivation

**Figure 3:**
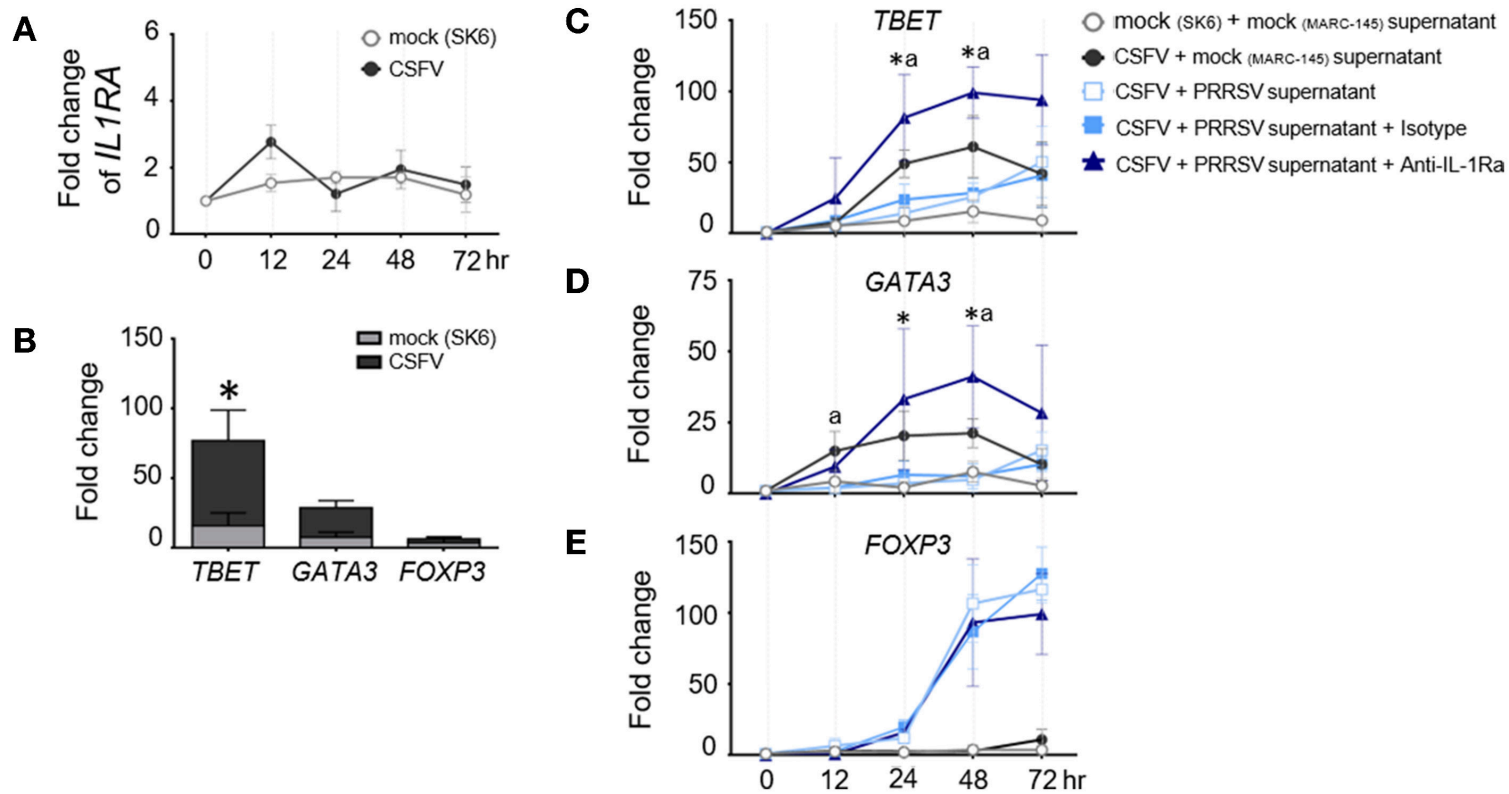
PRRSV-induced IL-1Ra altered the expression of helper T lymphocyte transcriptional factors during the recalled antigen test. **(A)** CSFV had no effect on *IL1RA* gene expression. **(B)** Upon activation with the recalled antigen, CSFV, strong upregulation of *TBET* was observed in the CSFV-primed PBMC. *indicates significant difference levels at *p* < 0.05. **(C–E)** The levels of transcriptional factor gene expression in the CSFV-primed PBMC, upon reactivation with the recalled antigen. PRRSV-induced IL-1Ra decreased expressions of **(C)** *TBET* and **(D)** *GATA3*, **(E)** but not *FOXP3* gene expressions. The supernatants from type 2 PRRSV- or mock (MARC-145 cell lysate)-infected MoDC were pretreated with anti-IL-1Ra Ab for 2 h prior to addition into the culture system. PBMC obtained from CSFV-immunized pigs were *in vitro* reactivated with CSFV or mock (SK6 cell lysate), in the presence of pretreated supernatants. Data represents mean ± SD from 5 pigs. Statistical significances were analyzed using *t* test **(A)** ANOVA followed by Tukey's test **(B–E)**. ^a^ indicates significant difference between mock-infected cell supernatant (black circle) and PRRSV-infected cell supernatants (close and open light blue squares) at *p* < 0.05. * indicates significant difference between addition of anti-IL-1Ra Ab (dark blue triangle) and PRRSV-infected cell supernatants (close and open light blue squares) at *p* < 0.05.

Development of porcine specific T lymphocyte lineages, namely Th1, Th2, and Treg, are specifically induced by constitutive expression of the transcription factors (TF); T-bet, GATA3, and FOXP3, respectively (36). It was previously reported that PRRSV infection interfered with host specific immune responses against other viral infections, including classical swine fever (CSFV) (44–46). Consequently, we investigated the immunomodulatory effect of PRRSV-induced IL-1Ra on the expression of major transcriptional factors during the recalled antigen (CSFV) responses. First, we confirmed that CSFV infection did not upregulate *IL1RA* gene expression in the CSFV-primed PBMC throughout the observation period (Figure 3A). Consistent with previous findings (47, 48), restimulation of CSFV-primed PBMCs with the same antigen significantly upregulated *TBET* expression indicating a shift of Th polarization toward Th1 (Figure 3B). Addition of the supernatant from PRRSV-infected MoDC decreased levels of CSFV-induced *TBET* and *GATA3* gene expression. The IL-1Ra neutralization was shown to restore or even increase the expressions of *TBET* and *GATA3* compared with CSFV restimulation alone (Figures 3C,D). Whereas, CSFV had no effect on *FOXP3* expression, the presence of supernatant of PRRSV-infected MoDC drastically upregulated transcriptional *FOXP3* at 48 and 72 h in CSFV-restimulated cells (Figures 3B,**E**). However, neutralization of IL-1Ra had no effect on the level of *FOXP3* expression (Figure 3E), suggesting Treg are differentiated by other mechanisms. Altogether, these results demonstrated the immunomodulatory effects of PRRSV-induced IL-1Ra on the antigen-specific Th differentiation.

### PRRSV-Induced IL-1Ra Inhibited Lymphocyte Proliferation, but Not IFN-γ Production

**Figure 4:**
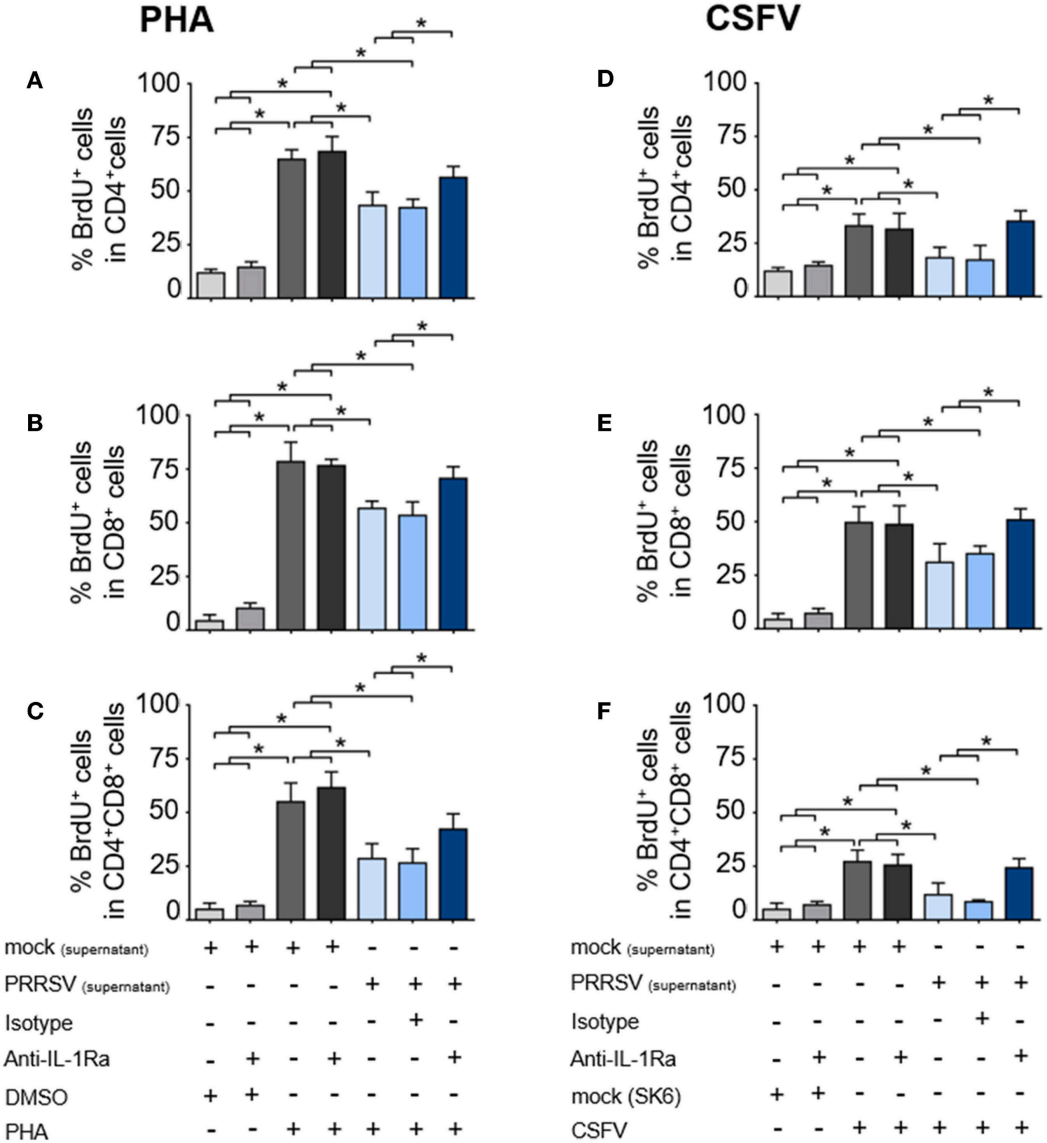
PRRSV-induced IL-1Ra inhibited lymphocyte proliferation. PRRSV-induced IL-1Ra inhibited PHA-induced proliferation of the **(A)** CD4^+^, **(B)** CD8^+^, and **(C)** CD4^+^CD8^+^ subpopulations. PRRSV-induced IL-1Ra inhibited CSFV-specific proliferations of the **(D)** CD4^+^, **(E)** CD8^+^, and **(F)** CD4^+^CD8^+^ subpopulations. The supernatants obtained from type 2 PRRSV or mock (MARC-145 cell lysate) were pretreated with anti-IL-1Ra Ab for 2 h prior to addition into the culture. PBL or PBMC were culture with PHA, CSFV or controls for 96 h, in the presence of the pretreated supernatants. ± indicates presence/absence of indicated treatment within the culture. Data represents mean ± SD from 5 pigs. Statistical significance was analyzed using ANOVA followed by Tukey's test. * indicates significant difference at *p* < 0.05.

To investigate the effect of PRRSV-induced IL-1Ra on T lymphocyte proliferation, the supernatants obtained from PRRSV-infected MoDC were pretreated with anti-IL-1Ra Ab, and then added into PHA- or CSFV-stimulated BrdU-cultured porcine PBL or PBMC. Lymphocytes were further gated into CD4^+^ (putative T helper lymphocytes), CD8^+^ (putative cytotoxic T lymphocytes) and CD4^+^CD8^+^ (putative memory T lymphocytes) subpopulations (Supplementary Figures 3A,B). Addition of PHA resulted in robust proliferation of CD4^+^, CD8^+^, and CD4^+^CD8^+^ subpopulations. Addition of anti-IL-1Ra Ab or isotype control did not affect PHA-stimulated cellular proliferation. On the contrary, the presence of PRRSV-infected MoDC supernatant significantly reduced the numbers of proliferating CD4^+^, CD8^+^, and CD4^+^CD8^+^ cells in the PHA-stimulated cultures, and these effects could be abrogated by addition of anti-IL-1Ra Ab (Figures 4A–C and Supplementary Figure 3A). The results indicated that PRRSV-induced IL-1Ra was involved in suppression of PHA-induced T lymphocyte proliferation.

Next, we examined the effect of PRRSV-induced IL-1Ra on antigen-specific T cell proliferation. Consistent with the above findings, the presence of CSFV induced antigen-specific proliferation of CD4^+^, CD8^+^, and CD4^+^CD8^+^ subpopulations from the CSFV-primed pigs. The CSFV-specific lymphocyte proliferation was significantly reduced in the presence of the supernatant obtained from the PRRSV-infected MoDC, whereas addition of anti-IL-1Ra Ab could restore the CSFV-specific lymphocyte proliferation (Figures 4D–F and Supplementary Figure 3B). The findings suggested the negative effect of PRRSV-induced IL-1Ra on porcine antigen-specific lymphocyte proliferation.

**Figure 5:**
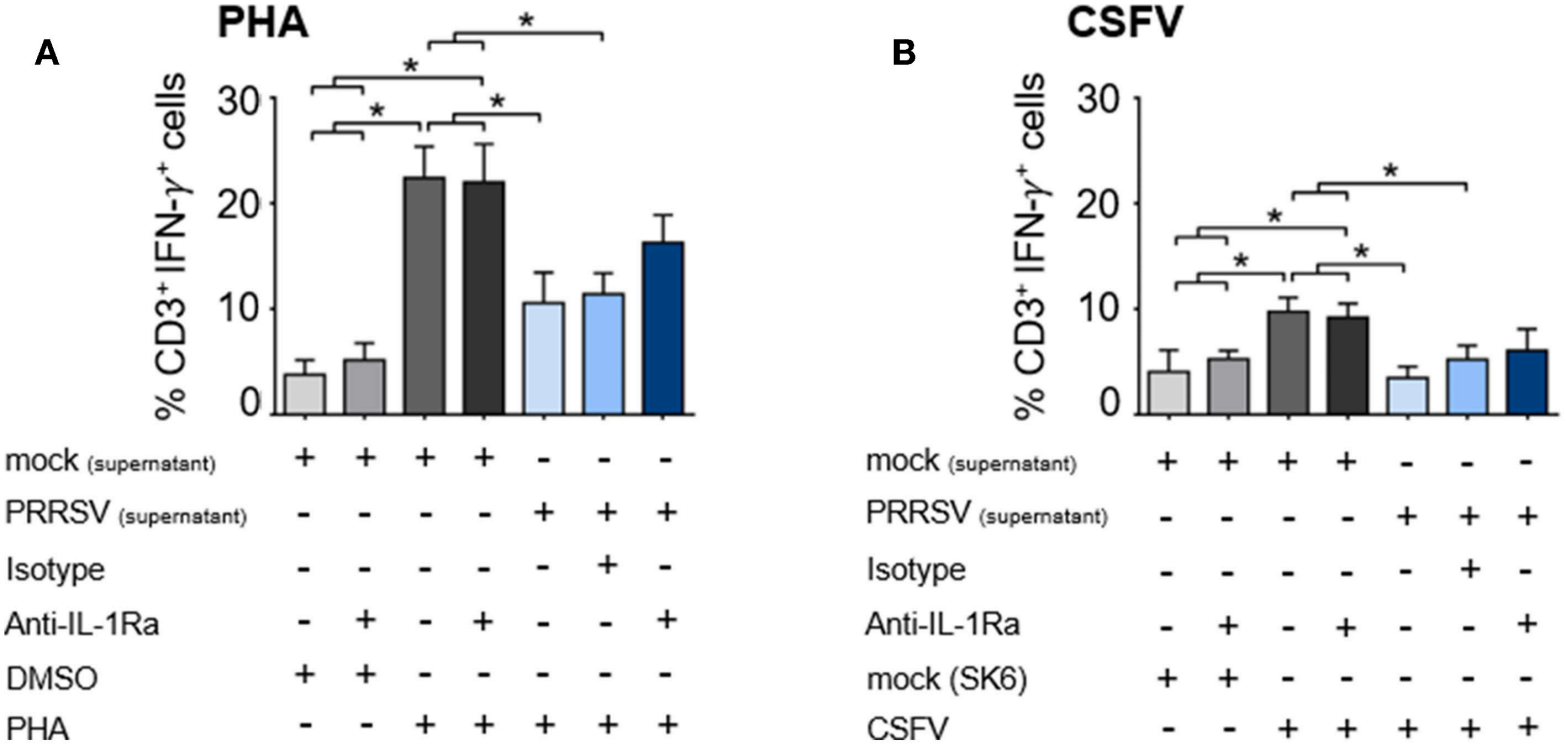
PRRSV-induced IL-1Ra was not involved in suppression of IFN-γ-producing T lymphocytes in both **(A)** polyclonal and **(B)** recalled CSFV responses. The supernatants obtained from type 2 PRRSV or mock (MARC-145 cell lysate) were pretreated with anti-IL-1Ra Ab for 2 h prior to addition into the culture. PBL or PBMC were cultured with PHA, CSFV or controls for 48 h, in the presence of the pretreated supernatants. ± indicates presence/absence of indicated treatment within the culture. Data represents mean ± SD from 5 pigs. Statistical significance was analyzed using ANOVA followed by Tukey's test. * indicates significant difference at *p* < 0.05.

We further examined the effect of PRRSV-induced IL-1Ra on T lymphocyte effector function by enumerating the number of IFN-γ-producing T lymphocytes. Addition of supernatant from PRRSV-infected MoDC significantly reduced the numbers of PHA-activated and CSFV-specific IFN-γ-producing T lymphocytes (Figures 5A,B and Supplementary Figures 5A,B). Addition of anti-IL-1Ra Ab into PHA-induced PBMC could only partially restore the numbers of IFN-γ-producing T lymphocytes (Figure 5A and Supplementary Figure 5A). Similarly, addition of anti-IL-1Ra Ab did not restore the numbers of CSFV-specific IFN-γ-producing cells in the culture system (Figure 5B and Supplementary Figure 5B). Together, our data demonstrated that the presence of PRRSV-induced IL-1Ra in the culture system significantly inhibited mitogen-induced and virus-specific lymphocyte proliferation. However, the negative effect of PRRSV-induced IL-1Ra on IFN-γ produced by T lymphocytes was not significant.

### PRRSV-Induced IL-1Ra Was Involved in the Induction of Treg, but Not IL-10-Producing T Lymphocytes

**Figure 6:**
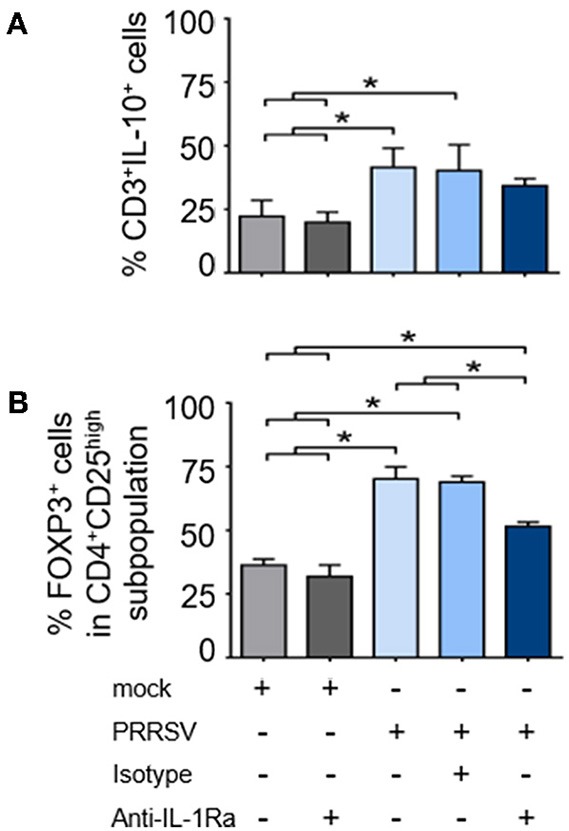
PRRSV-induced IL-1Ra was partially involved in regulatory T lymphocyte (Treg) induction. PRRSV-induced IL-1Ra decreased numbers of **(A)** Treg, **(B)** but not IL-10-producing T lymphocytes. PBMC were cultured in the presence of type 2 PRRSV or mock. Subsequently, anti-IL-1Ra Ab was added into the culture and further incubated for 48 h. ± indicates presence/absence of indicated treatment within the culture. Data represents mean ± SD from 5 pigs. Statistical significance was analyzed using ANOVA followed by Tukey's test. * indicates significant difference at *p* < 0.05.

As shown earlier, strong upregulation of *FOXP3* was observed in the presence of PRRSV-infected MoDC supernatant. To further investigate the role of PRRSV-induced IL-1Ra on IL-10 production and induction of Treg, PBMC were infected with PRRSV or mock, and in the presence or absence of anti-IL-1Ra Ab. Similarly to MoDC, PRRSV infection significantly induced IL-1Ra production in the cultured PBMC (Supplementary Figures 4A,B). In agreement with previous findings (23, 49, 50), PRRSV enhanced the numbers of IL-10-producing T lymphocytes and Treg (Figures 6A,B and Supplementary Figures 6A,B). Addition of anti-IL-1Ra Ab had little effect on the reduction of IL-10-producing T lymphocytes (Figure 6A and Supplementary Figure 6A). IL-1Ra neutralization significantly decreased the numbers of Treg, although not to the level of uninfected PBMC (Figure 6B and Supplementary Figure 6B). These findings indicated that PRRSV-induced IL-1Ra might not be directly involved in the development of IL-10-producing T lymphocytes, but could partly play a role in Treg induction under our studied conditions.

## Discussion

Interleukin-1 receptor antagonist (IL-1Ra) is known as an early inhibitory cytokine, which potentially participates in PRRSV-induced immunosuppression during the early phase of infection (26). As several reports showed that IL-1Ra is a potent negative immunomodulator of both innate and adaptive immune functions (51–53), the present study extended the characterization of the negative immunomodulatory effects of PRRSV-induced IL-1Ra on several aspects of porcine immune functions.

Porcine dendritic cell (DC) populations, i.e., conventional DC (cDC) and non-conventional DC, exhibit different PRRSV susceptibility. Several studies strongly indicated that PRRSV could not infect both cDC1 and cDC2 populations (54–56). It should be emphasized that this study utilized MoDC, a non-conventional DC, and that the observed PRRSV infectivity in porcine MoDC was consistent with the previous reports (10, 57). The immunomodulatory effects of PRRSV-induced IL-1Ra in the cDC population remains to be elucidated. In addition, variation in PRRSV infectivity of porcine myeloid cells might be related to the PRRSV strains used in the studies (58, 59).

The inhibition of phagocytic activity and APC maturation by PRRSV-induced IL-1Ra strongly supported the role of IL-1Ra in PRRSV immunopathogenesis. These findings were in agreement with the previous studies in human and mouse models reporting the suppressive effects of exogenous IL-1Ra on Fc-dependent phagocytosis (60), and APC maturation (52). Our observations that PRRSV-induced IL-1Ra downregulated *IFNA* and *IL1* gene expression were in agreement with previously published data on the inhibitory effects of IL-1Ra on type I IFN and pro-inflammatory cytokine productions in macrophages and DC (28, 61). Interestingly, suppression of *IL6* gene expression by PRRSV was not dependent with IL-1Ra under our experimental conditions. It must be pointed out that in experimental models of acute-phase response (62), IL-1Ra could not prevent excessive IL-6 production within local injury. Hence, our results suggested that other mechanisms besides IL-1Ra mediate PRRSV-suppressed IL-6 production.

PRRSV infection has long been shown to cause a negative impact on host immunity against other pathogens, including CSFV (44, 45). Differentiation into porcine T helper 1 (Th1) and T helper 2 (Th2) lymphocyte lineages requires specific expression of transcription factors (TF), TBET and GATA3, respectively (63, 64). Here, we demonstrated that PRRSV-induced IL-1Ra interfered with antigen-specific Th1/Th2 response by the downregulation of *TBET* and *GATA3* expressions. Supporting our findings, blocking of IL-1R signaling pathway inhibited Th1 and Th2 differentiations (53, 65). Surprisingly, IL-1Ra neutralization resulted in a higher level of *TBET* and *GATA3* expressions than those from the cells cultured with CSFV alone suggesting that a basal level of IL-1Ra is requested to balance the Th differentiation (66–68). Suppression of T lymphocyte differentiation by PRRSV-induced IL-1Ra most likely contributed to the observed negative impact of PRRSV on CSFV-specific immune responses in pigs (44, 45). We also demonstrated that PRRSV-induced IL-1Ra significantly suppressed mitogen- as well as antigen-dependent lymphocyte proliferation. The effect of PRRSV-induced IL-1Ra on T lymphocyte proliferation was quite expected as signaling via IL-1R was shown to be a prerequisite for induction of T helper lymphocyte (Th) proliferation (53). Moreover, blocking IL-1R activation with exogenous IL-1Ra could inhibit DNA replication, resulting in a reduction of T lymphocyte expansion (69, 70). Altogether, our findings suggested that the poor adaptive immune response, usually observed in PRRSV infected pigs, is in part due to the inhibition of T lymphocyte differentiation and proliferation by PRRSV-induced IL-1Ra.

The effector function of Th lymphocyte responses is attributable to specific types of cytokine production (71, 72). IFN-γ produced by Th1 lymphocytes can facilitate phagocytosis, antigen processing and presentation, antiviral stage of affected cells, and activation of effector cytotoxic T lymphocytes (CTL) (73). Delayed PRRSV-specific IFN-γ-secreting T lymphocyte response is usually observed during PRRSV infection (19, 74). However, transcriptomic analysis of PRRSV-immunized pigs revealed that the IFN-γ pathway was essential for activation of anti-viral defense mechanism (75). On the other hand, IL-10, a potent inhibitory cytokine, is known as one of the major PRRSV-induced immunosuppressive mechanisms (22, 49, 50). Consistent with previous findings, we demonstrated that PRRSV infection suppressed IFN-γ and increased IL-10-producing T lymphocytes. Although IL-1R signaling was also reported to play an important role in T lymphocyte effector function (53, 76), neutralization of IL-1Ra had no effect on both IFN-γ and IL-10 production in the cultured T cell populations. These observations convincingly pointed out that PRRSV-induced IL-1Ra might not be directly involved in T lymphocyte effector functions, but rather may interfere with T cell induction phase as observed through alteration of TF gene and cellular proliferation. The findings also suggested that other undefined immunomodulatory mechanisms might be responsible for these observations. A possible mechanism is via IL-10, as it directly interferes with IFN-γ production in memory T lymphocytes (77, 78). Although IL-10 production by other leukocyte populations was not determined in this study, previous reports evidenced the secretion of IL-10 from PRRSV-infected macrophages and DC (9, 50). Collectively, IL-10 produced by various leukocyte subpopulations may help promote IL-1Ra production as IL-10 can enhance production of IL-1Ra by recruitment of NF-κB to IL-1Ra promotor in monocytes and macrophages (79). It is likely that both IL-1Ra and IL-10 act synergistically in PRRSV-induced immunosuppression.

Induction of Treg, the inhibitory Th lymphocytes, is one of the PRRSV-induced negative immunomodulatory mechanisms (49, 80, 81). Development and function of Treg are distinct among Th lymphocyte lineages (82). Although expression of FOXP3 plays an important role during Treg differentiation and development, FOXP3 *per se* is not sufficient to maintain Treg phenotype and their functions (83). Other costimulatory regulators including PD-L1, CTLA-4, IL-2, IL-10, and TGF-β, collectively contribute to stable Treg phenotype and suppressive activities (84, 85), neutralization of IL-1Ra did not modulate PRRSV-induced *FOXP3* gene expression in the cultured PBMC (Figure 3E), but it could partially reduce the induction of PRRSV-induced FOXP3^+^CD4^+^CD25^high^ subpopulation (Figure 6B). It is possible that PRRSV-induced IL-1Ra does not help to promote Treg development, but potentially participates in maintaining Treg characteristics in the culture system. The finding on effects of PRRSV-induced IL-1Ra on Treg induction is intriguing. Previously, it was clearly demonstrated that IL-10 was responsible for induction of PRRSV-specific Treg (22). Apart from IL-10, PRRSV-induced TGF-β production is also involved in differentiation of inducible Treg (80, 86–89). There were some studies reporting that IL-1Ra could enhance TGF-β production in leukocytes and somatic cells (90, 91). This finding could strengthen our hypothesis on the role of PRRSV-induced IL-1Ra in the maintenance of Treg population.

Several key questions remain unanswered but need to be deeply investigated in order to fully characterize the roles of IL-1Ra in PRRSV immunopathogenesis. First, what is the precise molecular mechanism(s) of PRRSV-induced IL-1Ra in the infected cell? Second, what is the effect of PRRSV-induced IL-1Ra on IL-10 production, and vice versa, in myeloid cell population? Is it possible that both cytokines act synergistically to provide an immunological niche promoting Treg? Furthermore, in the context of PRRSV vaccine development, promising strategies based on the reduction of both induced IL-1Ra and IL-10 should also be explored. Although our observations on the negative immunomodulatory effects of PRRSV-induced IL-1Ra were obtained using *in vitro* assays, these circumstances are likely relevant to PRRSV-induced immunosuppression in the infected pigs in regard to impaired innate cytokine production (16, 92), prolonged PRRSV-specific T helper and CTL induction (93), and enhanced PRRSV-specific Treg population (22, 24, 94). In contrast, highly pathogenic (HP)-PRRSV infection, which is deficient in its ability for IL-1Ra induction (26), causes robust innate cytokine production in an early phase of infection (95). These findings support our notion that IL-1Ra is, in part, responsible for negative immune responses in the PRRSV-infected pigs.

In conclusion, this study highlighted the negative immunomodulatory effects of PRRSV-induced IL-1Ra on both porcine innate and adaptive immune functions. Our findings clearly demonstrated the inhibitory effects of PRRSV-induced IL-1Ra on APC functions, including phagocytosis and maturation, as well as interfering with the induction phase of T lymphocyte responses. Furthermore, IL-1Ra could help to maintain PRRSV-specific Treg population. This study confirms the role of IL-1Ra as a key immunosuppressor during the course of PRRSV infection.

## Data Availability

The datasets generated for this study are available on request to the corresponding author.

## Ethics Statement

Animal care and use protocols for this study followed the *Ethical Principles and Guidelines for the Use of Animals*, National Research Council of Thailand, and the *Guide for the Care and Use of Laboratory Animals*, National Research Council, USA. All methods and animal studies were approved by Chulalongkorn University Animal Care and Use Committee, Chulalongkorn University (Animal Use Protocol No. 1631029).

## Author Contributions

TN, NT, RT, and SS designed research studies. TN and NT performed experiments, acquired data, analyzed data. TN, NT, and SS drafted the manuscript.

### Conflict of Interest Statement

The authors declare that the research was conducted in the absence of any commercial or financial relationships that could be construed as a potential conflict of interest.
