# Supplementary material for: Negative Immunomodulatory Effects of Type 2 Porcine Reproductive and Respiratory Syndrome Virus-Induced Interleukin-1 Receptor Antagonist on Porcine Innate and Adaptive Immune Functions
Source: Front Immunol. 2019 Mar 26;10:579. doi: 10.3389/fimmu.2019.00579 (PMC6443931; doi:10.3389/fimmu.2019.00579)
Supplement: Supplementary file 1 [file Data_Sheet_1.docx]

Supplementary Material

**Negative immunomodulatory effects of type 2 porcine reproductive and respiratory syndrome virus-induced interleukin-1 receptor antagonist on porcine innate and adaptive immune functions**

Teerawut Nedumpun, Navapon Techakriengkrai, Roongroje Thanawongnuwech,

Sanipa Suradhat^*^

***Correspondence:** Sanipa Suradhat: [Sanipa.S@chula.ac.th](mailto:Sanipa.S@chula.ac.th)

## Supplementary Figures


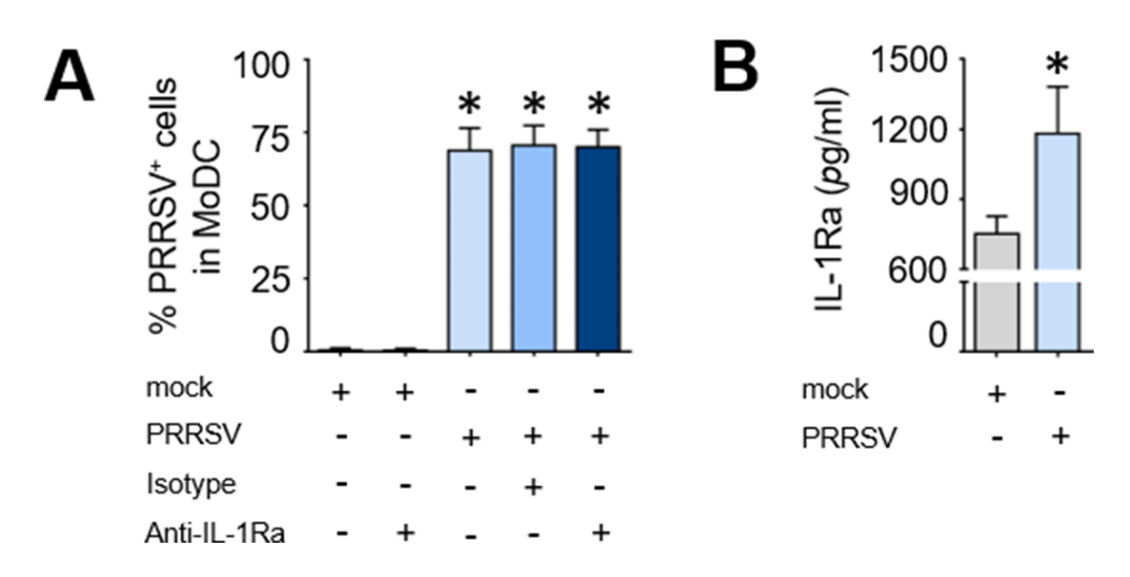


**Supplementary Figure 1.** The percentage of PRRSV-infected cells **(A)** and level of IL-1Ra production **(B)** in the PRRSV-infected MoDC. MoDC were cultured with 0.1 m.o.i. of type 2 PRRSV or MARC-145 cell lysate (mock) for 48 hr. Secretory IL-1Ra in the supernatants and number of PRRSV-infected cells were quantified by ELISA and flow cytometry, respectively. + indicates the presence of each treatment within the culture. Data represent mean±SD from 5 pigs. Statistical significances were analyzed using ANOVA followed by Tukey’s test (**A**) and *t* test (**B**). * indicates a significant difference between the treatment and other controls (mock) at *p*<0.05.

**^
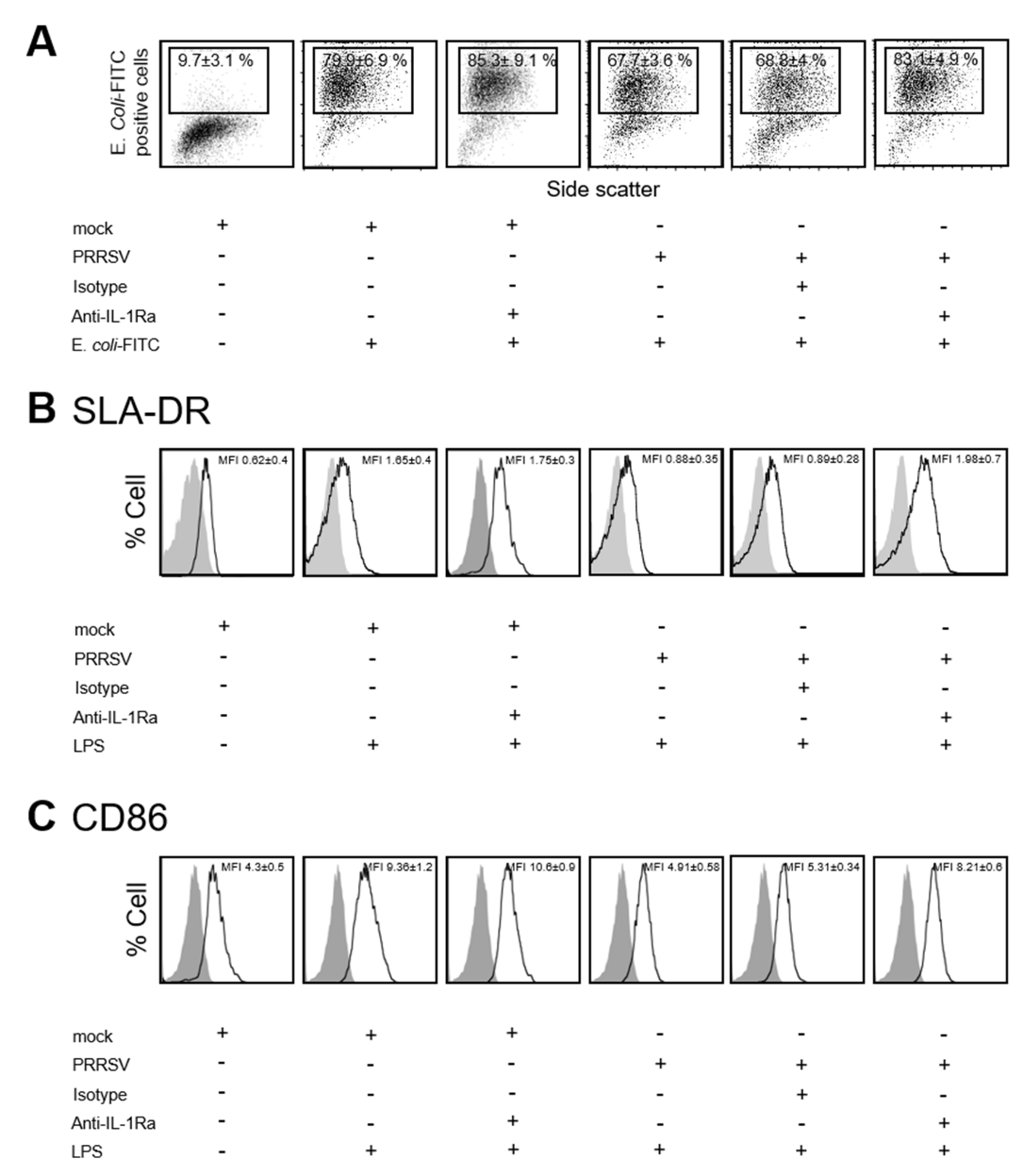
^**

**Supplementary Figure 2.** PRRSV-induced IL-1Ra inhibited porcine innate immune functions. PRRSV-induced IL-1Ra inhibited **(A)** phagocytic activity, **(B)** SLA-DR and **(C)** CD86 expressions. MoDC were cultured with type 2 PRRSV and LPS. Subsequently, anti-IL-1Ra mAb was added into the culture and further incubated for 24 hr. +/- indicates the presence/absence of indicated treatment within the culture.


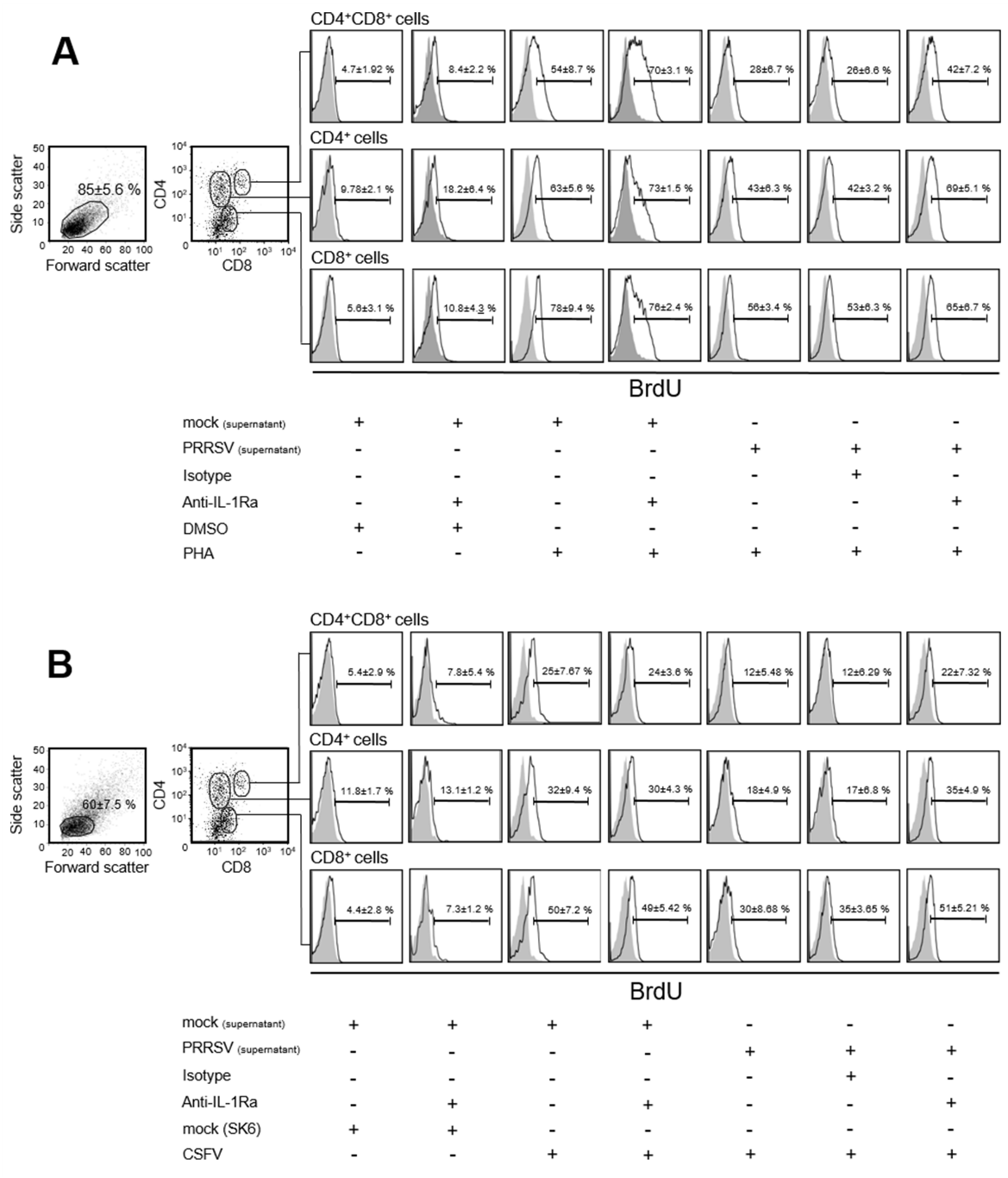


**Supplementary Figure 3.**  PRRSV-infected IL-1Ra inhibited lymphocyte proliferation. **(A-B)** The cells were gated into CD4^+^CD8^+^, CD4^+^ and CD8^+^ subpopulations and further analyzed the proliferative characteristic (BrdU^+^ cells). PRRSV-induced IL-1Ra involved in suppressions of **(A)** polyclonal and **(B)** CSFV-specific lymphocyte proliferation. The supernatants obtained from type 2 PRRSV or mock (MARC-145 cell lysate) were pretreated with anti-IL-1Ra mAb for 2 hr prior to addition into the culture. PBL or PBMC were cultured in the presence of the pretreated supernatants and PHA, CSFV or controls for 96 hr. +/- indicates the presence/absence of indicated treatment within the culture.

**
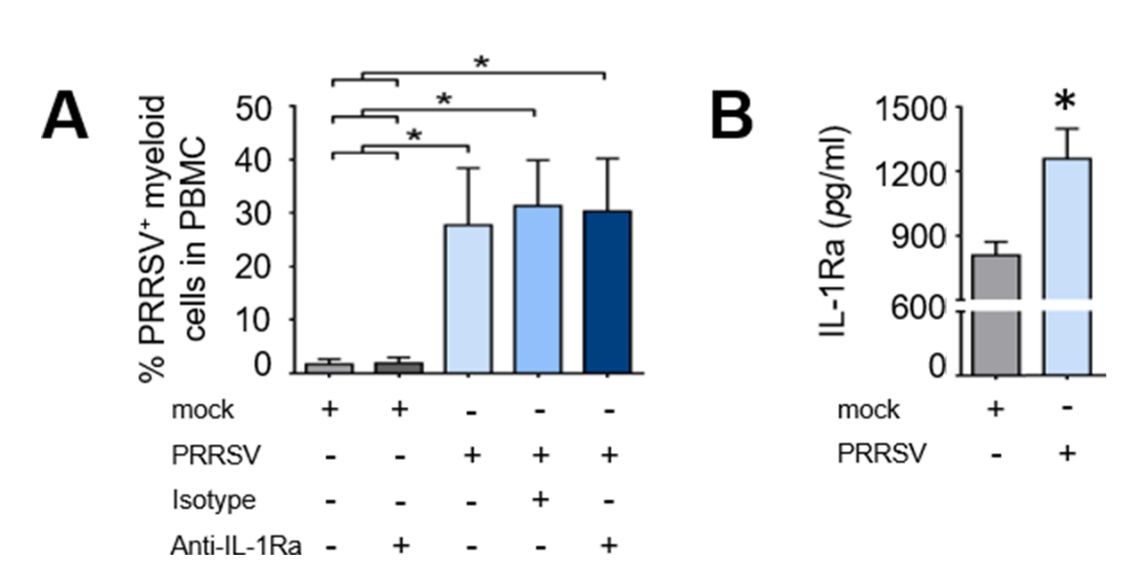
**

**Supplementary Figure 4.** Percentages of PRRSV-infected myeloid cells **(A)** and levels of IL-1Ra production **(B)** in the cultured PBMC. The PBMC were cultured with 0.1 m.o.i. of type 2 PRRSV or MARC-145 cell lysate (mock) for 48 hr. The number of PRRSV-infected cells and level of IL-1Ra production in the culture were quantified using flow cytometry and ELISA, respectively. +/- indicates the presence/absence of indicated treatment within the culture. Data represents mean±SD from 5 pigs. Statistical significances were analyzed using ANOVA followed by Tukey’s test (**A**) and *t* test (**B**). * indicates a significant difference between the treatment and other controls (mock) at *p*<0.05.

**
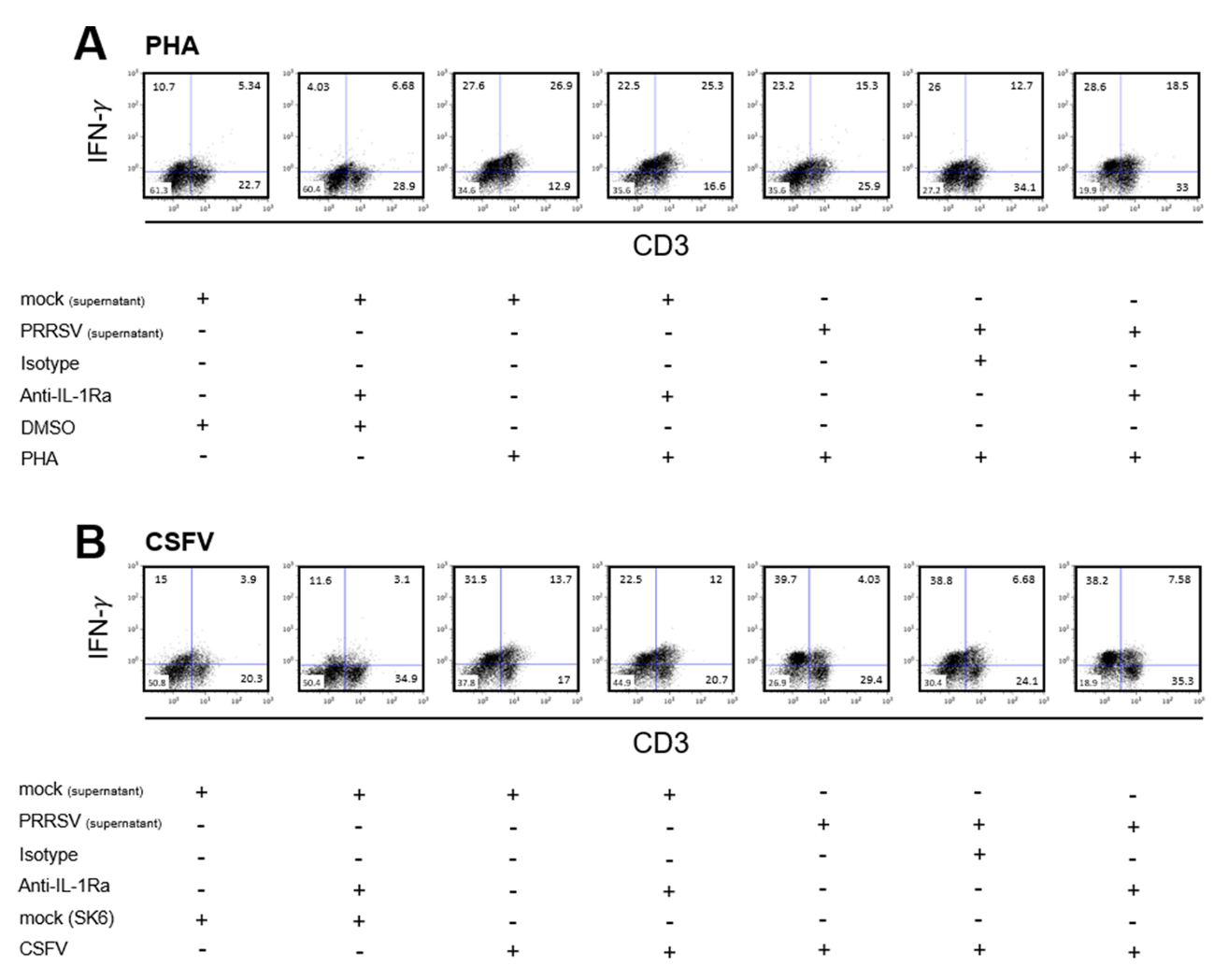
**

**Supplementary Figure 5.** PRRSV-induced IL-1Ra was not involved in the suppression of IFN-γ-producing T lymphocytes in both (**A**) polyclonal and (**B**) recalled CSFV responses. The supernatants obtained from type 2 PRRSV or mock (MARC-145 cell lysate) were pretreated with anti-IL-1Ra mAb for 2 hr prior to addition into the culture. PBL or PBMC were culture with PHA, CSFV or controls for 48 hr, in the presence of the pretreated supernatants. +/- indicates the presence/absence of indicated treatment within the culture.


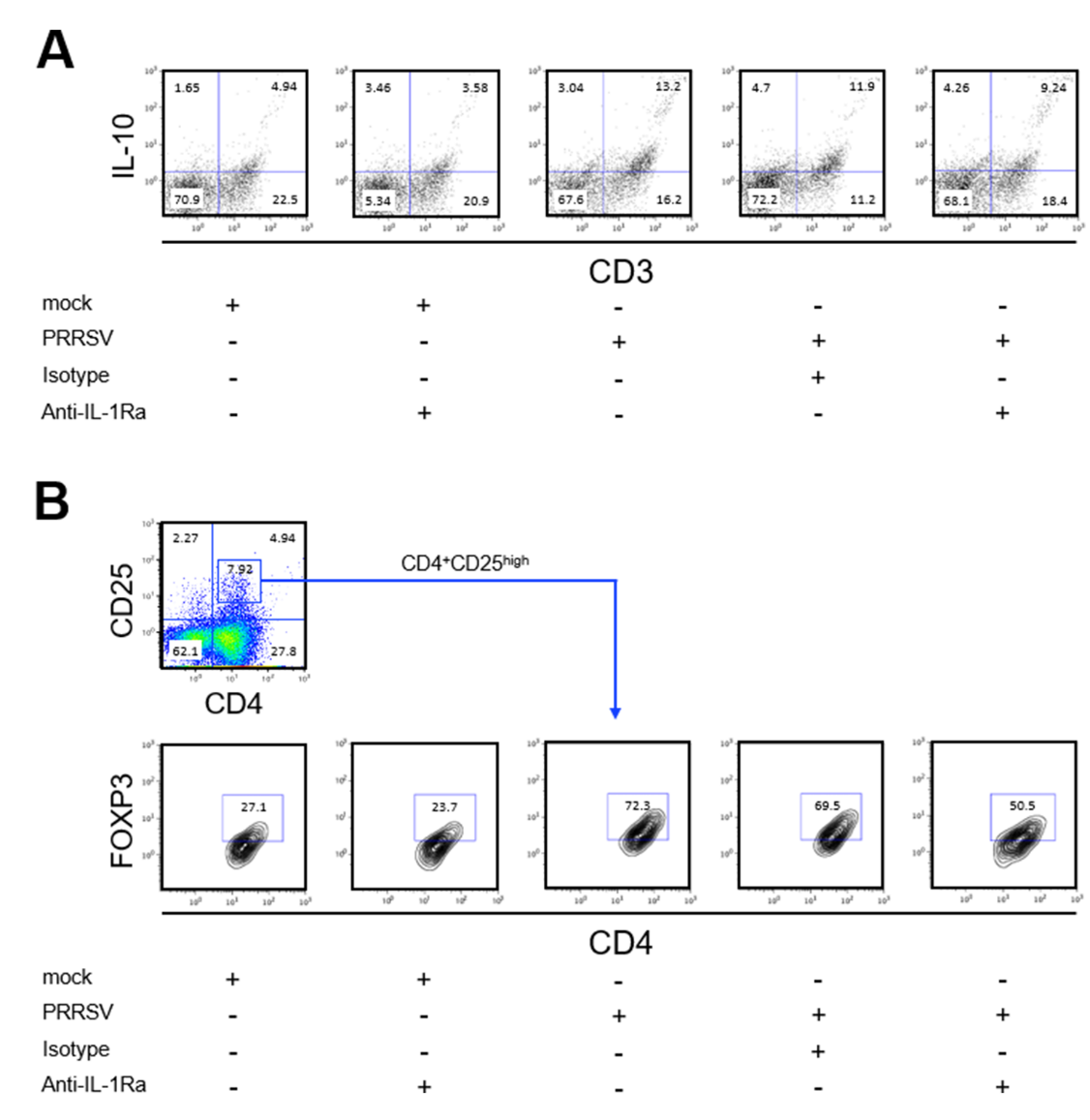


**Supplementary Figure 6.** PRRSV-induced IL-1Ra was partially involved in regulatory T lymphocytes (Treg) induction. PRRSV-induced IL-1Ra decreased numbers of (**A**) Treg, (**B**) but not IL-10-producing T lymphocytes. PBMC were cultured in the presence of type 2 PRRSV or mock. Subsequently, anti-IL-1Ra mAb was added into the culture and further incubated for 48 hr. +/- indicates the presence/absence of indicated treatment within the culture.
